# Supplementary material for: Integrated Collection of Stem Cell Bank Data, a Data Portal for Standardized Stem Cell Information
Source: Stem Cell Reports. 2021 Mar 18;16(4):997–1005. doi: 10.1016/j.stemcr.2021.02.014 (PMC8072026; doi:10.1016/j.stemcr.2021.02.014)
Supplement: Document S1. Supplemental Experimental Procedures, Figures S1–S3, and Tables S2–S5 [file mmc1.pdf]

Stem Cell Reports, Volume *16*

Supplemental Information

Integrated Collection of Stem Cell Bank Data, a Data Portal for Standardized Stem Cell Information

Ying Chen, Kunie Sakurai, Sumihiro Maeda, Tohru Masui, Hideyuki Okano, Johannes Dewender, Stefanie Seltmann, Andreas Kurtz, Hiroshi Masuya, Yukio Nakamura, Michael Sheldon, Juliane Schneider, Glyn N. Stacey, Yulia Panina, and Wataru Fujibuchi

SUPPLEMENTAL FIGURES

Fig S1. Related to Figure 2.

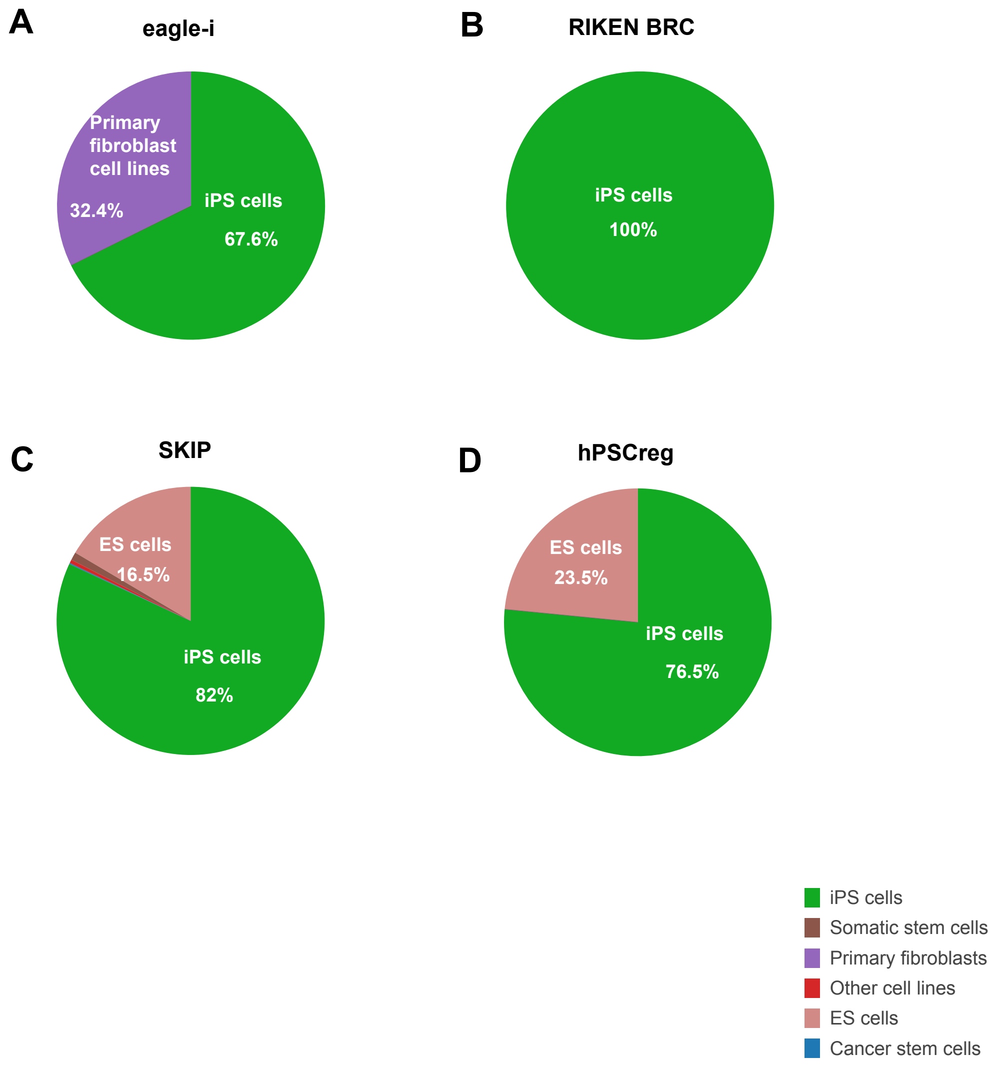

Fig S1. Details of cell line types collected by eagle-i, RIKEN BRC, SKIP, and hPSCreg (as of December 6, 2020)**.** (A) eagle-i, (B) RIKEN BRC, (C) SKIP, and (D) hPSCreg.

Fig S2. Related to Figure 2.

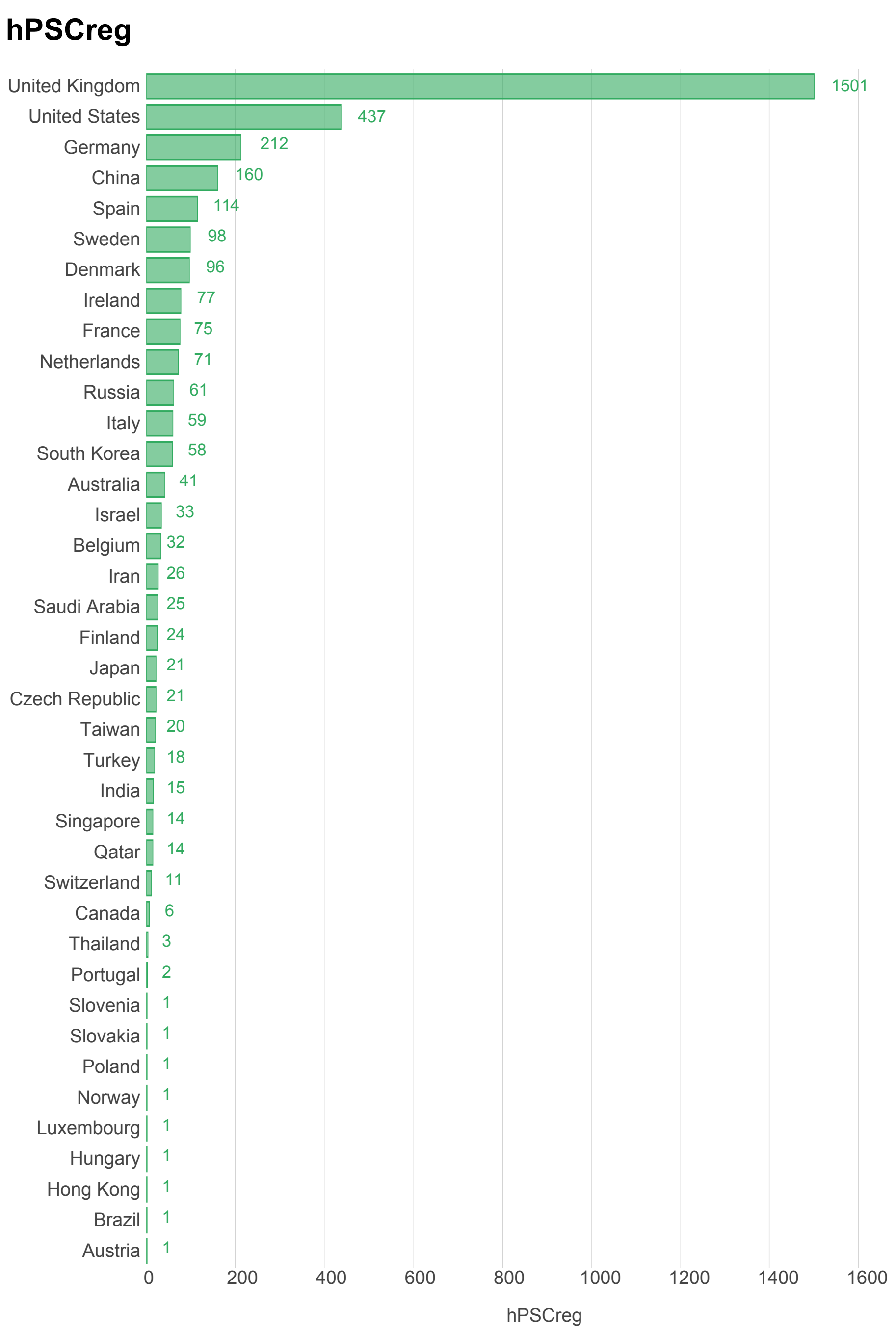

Fig S2. **Details of countries that have established cell lines in hPSCreg** (as of December 6, 2020)**.**

Fig S3. Related to Figure 2.

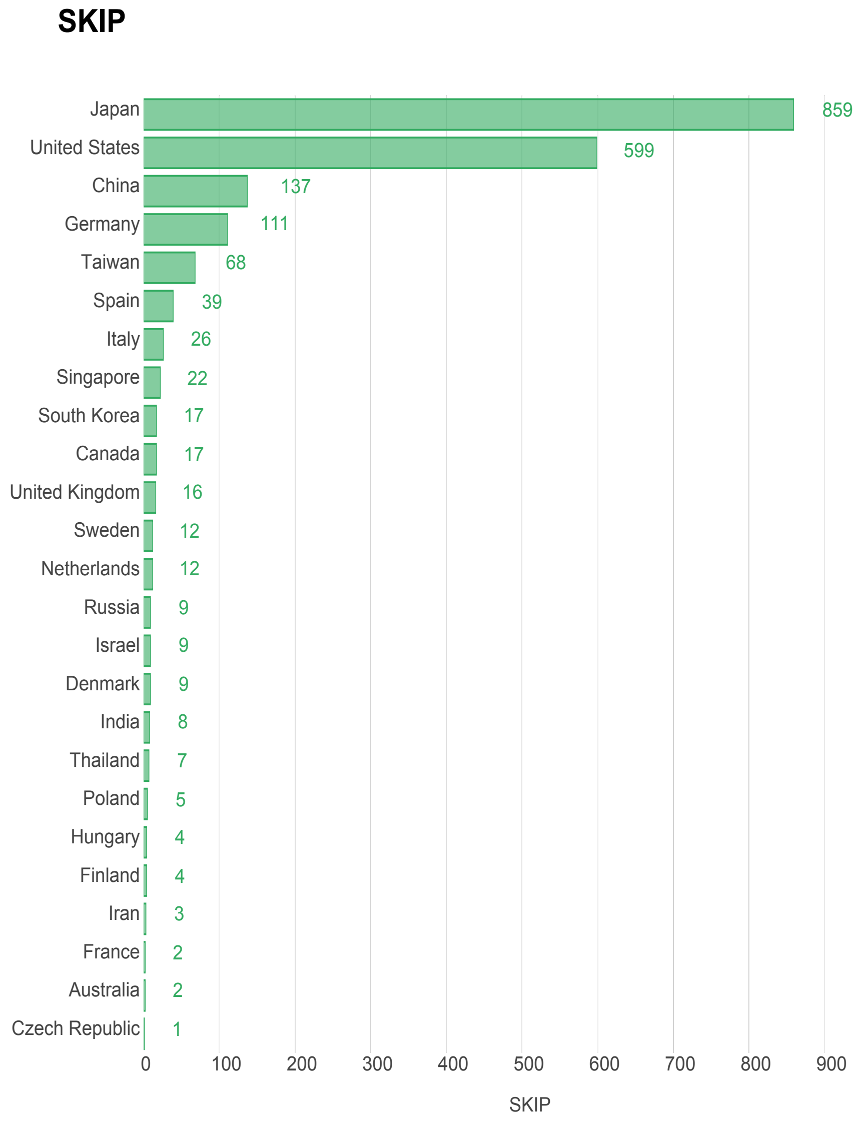

Fig S3. Details of countries that have established cell lines in SKIP (as of December 6, 2020)**.**

SUPPLEMENTAL TABLES

Table S1. Related to Figure 5. (submitted as separate Excel file)

Table S1. List of full information of ICSCB (as of December 6, 2020)**.**

Table S2. Related to Figure 2A and Figure S1.

Stem_cell_type	hPSCreg	SKIP	RIKEN BRC
**ES cells**	788	927	0
**iPS cells**	2572	4603	3548
**Somatic stem cells**	0	54	0
**Cancer stem cells**	0	7	0
**Primary fibroblast cell lines**	0	0	0
**Others**	0	24	0
**Total**	**3360**	**5615**	**3548**

Table S2. List of cell line types across all four databases (as of December 6, 2020)**.**

Table S3. Related to Figure 2B.

Health_status	Count
Healthy	9708
Diseased	6363
**Total**	**16071**

Table S3. Statistics of healthy/diseased cell lines in ICSCB (as of December 6, 2020)**.**

Table S4. Related to Figure 2C.

Country	SKIP	hPSCreg	Total
**United Kingdom**	16	1501	1517
**United States**	599	437	1036
**Japan**	859	21	880
**Germany**	111	212	323
**China**	137	160	297
**Spain**	39	114	153
**Sweden**	12	98	110
**Denmark**	9	96	105
**Taiwan**	68	20	88
**Italy**	26	59	85
**Ireland**	0	77	77
**South Korea**	17	58	75
**Netherlands**	12	71	83
**Russia**	9	61	70
**France**	2	75	77
**Australia**	2	41	43
**Israel**	9	33	42
**Singapore**	22	14	36
**Belgium**	0	32	32
**Iran**	3	26	29
**Canada**	17	6	23
**Finland**	4	24	28
**Czech Republic**	1	21	22
**India**	8	15	23
**Turkey**	0	18	18
**Qatar**	0	14	14
**Switzerland**	0	11	11
**Thailand**	7	3	10
**Hungary**	4	1	5
**Poland**	5	1	6
**Portugal**	0	2	2
**Saudi Arabia**	0	25	25
**Austria**	0	1	1
**Brazil**	0	1	1
**Luxembourg**	0	1	1
**Norway**	0	1	1
**Slovakia**	0	1	1
**Slovenia**	0	1	1
**Hong Kong**	0	1	1
**Total**	**1998***	**3354****	**5352**

*The number was calculated by 5615 (total) - 3647 (hPSCreg or eagle-i or unknown country) + 30 (dual country) = 1998.

**The number was calculated by 3360 (total) - 6 (synonyms) = 3354

Table S4. Statistics of cell line types based on country (as of December 6, 2020)**.**

Table S5. Related to Figure 2D.

Disease Category in MeSH	Count of diseases
Urogenital Diseases	26
Skin and Connective Tissue Diseases	12
Others	16
Nutritional and Metabolic Diseases	21
Nervous System Diseases	614
Neoplasms	16
Musculoskeletal Diseases	52
Mental Disorders	90
Male Urogenital Diseases	25
Immune System Diseases	15
Hemic and Lymphatic Diseases	26
Genetic Disorders	179
Eye Diseases	52
Endocrine System Diseases	108
Digestive System Diseases	3
Chemically Induced Disorders	4
Cardiovascular Diseases	107
**Total**	**1366**

Table S5. Statistics of diseased cell lines based on disease category (as of December 6, 2020)**.**

EXPERIMENTAL PROCEDURES

Generation of Fig. 1

Among all the databases, SKIP and hPSCreg provided details of countries from which data were acquired. For SKIP, this information was provided on its homepage (skip.stemcellinformatics.org/en/). For hPSCreg, country information for every cell line could be accessed from its homepage (https://hpscreg.eu/) by clicking “find by location”.

Generation of Fig. 2

Full information data were directly downloaded from ICSCB results page (Table S1) and filtered according to the following criteria: (A) stem cell type (Table S2); (B) health/disease status (Table S3); (C) country (Table S4); and (D) disease (Table S5). Disease categories were determined by search results with keywords under the “Disease Category” in NCBI MeSH page (https://www.ncbi.nlm.nih.gov/mesh). For example, searching with keywords of “Parkinson disease” will lead to the MeSH term “Nervous System Diseases” under the “Disease Category”. Pie charts and bar graphs were produced by R (graph.r) using the package “plotly”.
