# Supplementary material for: Integrated Collection of Stem Cell Bank Data, a Data Portal for Standardized Stem Cell Information
Source: Stem Cell Reports. 2021 Mar 18;16(4):997–1005. doi: 10.1016/j.stemcr.2021.02.014 (PMC8072026; doi:10.1016/j.stemcr.2021.02.014)
Supplement: Document S2. Article plus Supplemental information [file mmc3.pdf]

Stem Cell Reports
Resource
OPEN ACCESS

Integrated Collection of Stem Cell Bank Data, a Data Portal for Standardized Stem Cell Information

Ying Chen,[1] Kunie Sakurai,[1,13] Sumihiro Maeda,[2] Tohru Masui,[3] Hideyuki Okano,[2] Johannes Dewender,[4] Stefanie Seltmann,[4] Andreas Kurtz,[4,5] Hiroshi Masuya,[6] Yukio Nakamura,[7] Michael Sheldon,[8] Juliane Schneider,[9] Glyn N. Stacey,[10,11,12] Yulia Panina,[1] and Wataru Fujibuchi[1,*]

[1]Center for iPS Cell Research and Application (CiRA), Kyoto University, 53 Kawahara-cho, Sho-goin, Sakyo-ku, Kyoto 606-8507, Japan
[2]Department of Physiology, Keio University School of Medicine, Tokyo 160-8582, Japan
[3]National Center for Medical Genetics, Keio University School of Medicine, Tokyo 160-8582, Japan
[4]Fraunhofer Institute for Biomedical Engineering, Biomedical Data and Bioethics, Anna-Louisa-Karsch-Strasse 2, 10178 Berlin, Germany
[5]BIH Center for Regenerative Therapies (BCRT), Charité–Universitätsmedizin Berlin, Augustenburger Platz 1, 13353 Berlin, Germany
[6]Integrated Bioresource Information Division, RIKEN BioResource Research Center, Tsukuba, Ibaraki 305-0074, Japan
[7]Cell Engineering Division, RIKEN BioResource Research Center, Tsukuba, Ibaraki 305-0074, Japan
[8]Department of Genetics and Human Genetics Institute of New Jersey, Rutgers, The State University of New Jersey, Piscataway, NJ 08854, USA
[9]Harvard Catalyst|The Harvard Clinical and Translational Science Center, Boston, MA 02215, USA
[10]International Stem Cell Banking Initiative, 2 High Street, Barley, Hertfordshire SG88HZ, UK
[11]National Stem Cell Resource Center, Institute of Zoology, Chinese Academy of Sciences, Beijing 100190, China
[12]Innovation Academy for Stem Cell and Regeneration, Chinese Academy of Sciences, Beijing 100101, China
[13]Present address: Department of Cellular Biology and Pharmacology, Herbert Wertheim College of Medicine, Florida International University, Miami, FL 33199, USA
*Correspondence: fujibuchi-g@cira.kyoto-u.ac.jp
https://doi.org/10.1016/j.stemcr.2021.02.014

SUMMARY

The past decade has witnessed an extremely rapid increase in the number of newly established stem cell lines. However, due to the lack of a standardized format, data exchange among stem cell line resources has been challenging, and no system can search all stem cell lines across resources worldwide. To solve this problem, we have developed the Integrated Collection of Stem Cell Bank data (ICSCB) (http://icscb.stemcellinformatics.org/), the largest database search portal for stem cell line information, based on the standardized data items and terms of the MIACARM framework. Currently, ICSCB can retrieve >16,000 cell lines from four major data resources in Europe, Japan, and the United States. ICSCB is automatically updated to provide the latest cell line information, and its integrative search helps users collect cell line information for over 1,000 diseases, including many rare diseases worldwide, which has been a formidable task, thereby distinguishing itself from other database search portals.

INTRODUCTION

Since the first report of human induced pluripotent stem cells (iPSCs) (Takahashi et al., 2007), there has been a rapid increase in the number of iPSC lines and related information worldwide (Table 1). This remarkable growth has not only accelerated studies of regenerative medicine but also provided opportunities to understand such pragmatic issues as the quality of pluripotent stem cells (Nishizawa et al., 2016) and the disease mechanisms (Sasaki et al., 2016). Stem cell banks and registries are expected to provide necessary data on individual stem cell lines. However, the exchange of data among institutions is not a trivial matter, and the scientific reproducibility of the stem cells, particularly iPSCs generated by different methods, depending on available information is problematic for both basic studies and clinical applications (Isasi and Knoppers, 2011; Thirumala et al., 2009; Yaffe et al., 2016). Moreover, as technologies for the characterization of cell lines continue to advance, the addition of new quality standards as necessary data items has complicated and diversified data formats among stem cell banks and registries (Hug, 2009; Knoppers and Isasi, 2010). As an attempt to solve these problems, we previously reported MIACARM (Minimum Information About a Cellular Assay for Regenerative Medicine) guidelines in 2016 (Sakurai et al., 2016), which proposed the utilization of standardized data items and formats for all stem cell lines in regenerative medicine. At present, MIACARM contains 260 items covering such areas as stem cell production and materials (e.g., donor information, source cell information, and cell culture medium and substrate information), cell banking processes, cell characterization, sterility testing, and even ethical concerns. Later, a standardized nomenclature for pluripotent stem cells was introduced in 2018 with unification of cell line codification and minimization of information loss and confusion regarding cell lines as goals (Kurtz et al., 2018). Nevertheless, with the growing number of registered cell lines, existing data deposition formats have made it increasingly harder for not only data depositors but also users to seek and obtain cell lines collected under different projects, disease states, and privacy issues (Godard et al., 2003; Winickoff et al., 2009).

This is an open access article under the CC BY license (http://creativecommons.org/licenses/by/4.0/).

Table 1. Stem Cell Banks and Registries Worldwide (as of December 6, 2020)

B/R[a]	Stem Cell Bank or Registry	Country	Website	Number of Cell Lines	Data Included in ICSCB's Registries (○, Included; △, Planned) SKIP	eagle-i	hPSCreg
B	BLCB	Spain	https://p-cmrc.cat/	176			
R	hPSCreg	Germany	https://hpscreg.eu/	3,360	○		○
B/R	HipSci	United Kingdom	http://www.hipsci.org/	3,720			○
B	EBiSC	Germany	https://ebisc.org/	897			○
B/R	CIRM/FufiFilm	United States	https://fujifilmcdi.com/the-cirm-ipsc-bank/	1,554			△
B	Harvard Stem Cell Institute	United States	http://stemcelldistribution.harvard.edu/	41			
B	NYSCF	United States	https://nyscf.org/	111		○	
B	NINDS Human Cell and Data Repository	United States	https://bioq.nindsgenetics.org/	162			
B	WiCell Research Institute	United States	https://www.wicell.org/	1,519		○	△
B/R	eagle-i	United States	https://www.eagle-i.net/	2,415	○	○	
B/R	RIKEN BRC	Japan	https://www.brc.riken.jp/en/	4,102	○		
R	SKIP	Japan	https://skip.stemcellinformatics.org/	5,770	○		
B	JCRB	Japan	https://cellbank.nibiohn.go.jp/	31	○		
B	Taiwan Human Disease iPSC Service Consortium	Taiwan	https://catalog.bcrc.firdi.org.tw/	102	○		
B	National Stem Cell Bank of Korea	Korea	http://kscr.nih.go.kr/	147			

[a]B, bank; R, registry.

In this paper, as our next step toward the unification and utilization of the stem cell line data in the world, we report our new database portal, Integrated Collection of Stem Cell Bank data (ICSCB), which was designed using MIACARM guideline items and formats to serve as an entrance "port" to individual data repositories. The main objectives of ICSCB are (1) to establish an integrated stem cell database portal that can cover the majority of stem cell resources in the world and (2) to offer users minimum but efficient access to information on stem cell lines based on MIACARM guidelines. Currently, ICSCB provides data on more than 15,000 stem cell lines registered in four major stem cell line databases: hPSCreg (Seltmann et al., 2015), SKIP (Kim et al., 2017), RIKEN BRC (Kobayashi et al., 2016), and eagle-i (Vasilevsky et al., 2012). ICSCB has a user-friendly search engine for stem cell lines and can be accessed directly at http://icscb.stemcellinformatics.org/ or, as a slim version by removing cell line redundancy as much as possible, through the SHOGoiN (Human Omics Database for the Generation of iPS and Normal Cells) homepage at http://shogoin.stemcellinformatics.org/.

RESULTS

Web Interface

ICSCB was designed for researchers searching for available cell lines to conduct various studies, such as regenerative medicine and disease analysis. Covering as many diverse cell lines as possible was the first priority when deciding which resources to include in ICSCB. Sharing cell line information between different stem cell banks and registries has been problematic due to different cell naming methods, different policies on cell assessment in different registries, unclear data sources, and so on. ICSCB is a collection of cell lines from four major and reliable cell line data resources

A

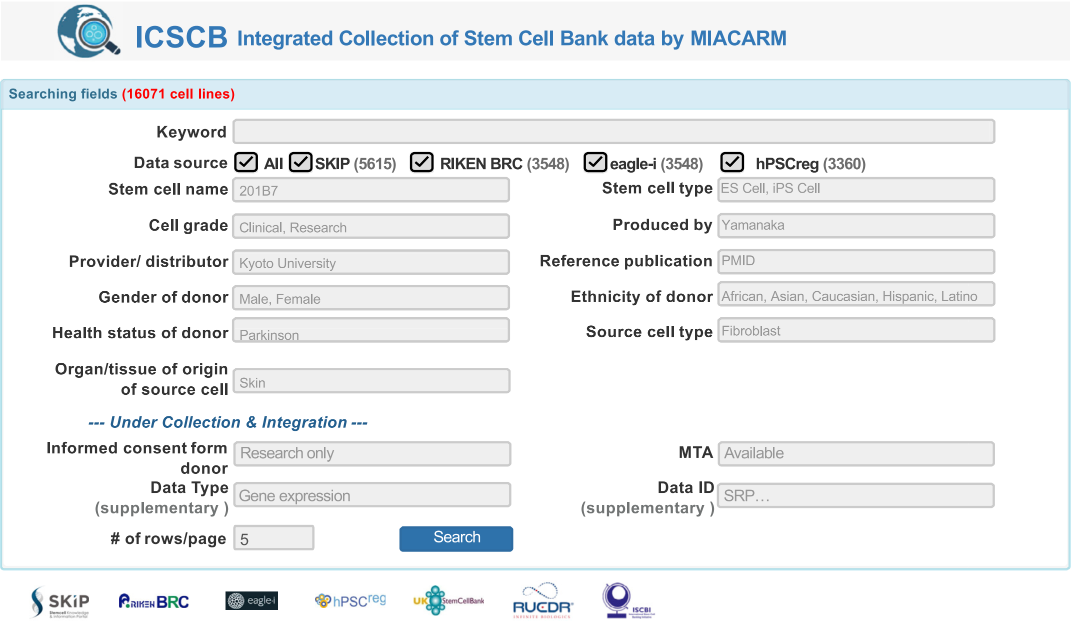

B

Data source	Source cell													Data	
	Stem cell production														
	Stem cell general identification						Donor identification			Source cell identification		Ethical operation			
	Stem cell ID	Stem cell name	Stem cell type	Cell grade	Produced by	Provider/ distributor	Reference publication	Gender of donor	Ethnicity of donor	Health status of donor	Source cell type	Organ/ tissue of origin of source cell	Informed consent from donor	MTA	Data ID	Data type
RIKEN BRC	HPS0045	HiPS-RIKEN-5A	Human iPS cell lines		Shimizu, Natsumi				Japanese	Wilson disease				Description: Disease specific iPS cell line derived from a patient : Wilson's disease (RCB0395 NCU-F8). Order Form (C-0042, C-0007 C-0007p).		

As of December 06, 2020

Figure 1. Web Interface of ICSCB

(A) The ICSCB search page. Any keyword related to cell lines (including cell line name, disease name, gender, and so on) can be used to perform an instant search.

(B) The ICSCB results page. Matched or partially matched cell lines are listed according to MIACARM terms. To check the details of the cell lines, the user can click on the stem cell ID, which is linked to the original source of cell line information.

based in Europe, Japan, and the United States. ICSCB updating is regularly performed for new SKIP and eagle-i stem cell line data as well as automatically performed for hPSCreg and RIKEN BRC data in a synchronized manner. Users can retrieve all related stem cell line information by using a free text search. Detailed information on a specific cell line can be accessed by clicking on the stem cell ID, which is linked to the information page in the original resources (Figure 1). The results can be further filtered according to users' requests. There may be several records for the same cell line if the cell line is included in multiple data resources. To provide users as much information as possible, the results page is designed to show cell lines with matching cell names as well as similar descriptions.

Data Coverage

ICSCB covers more data than any other stem cell line repository available. The integration of all major data resources

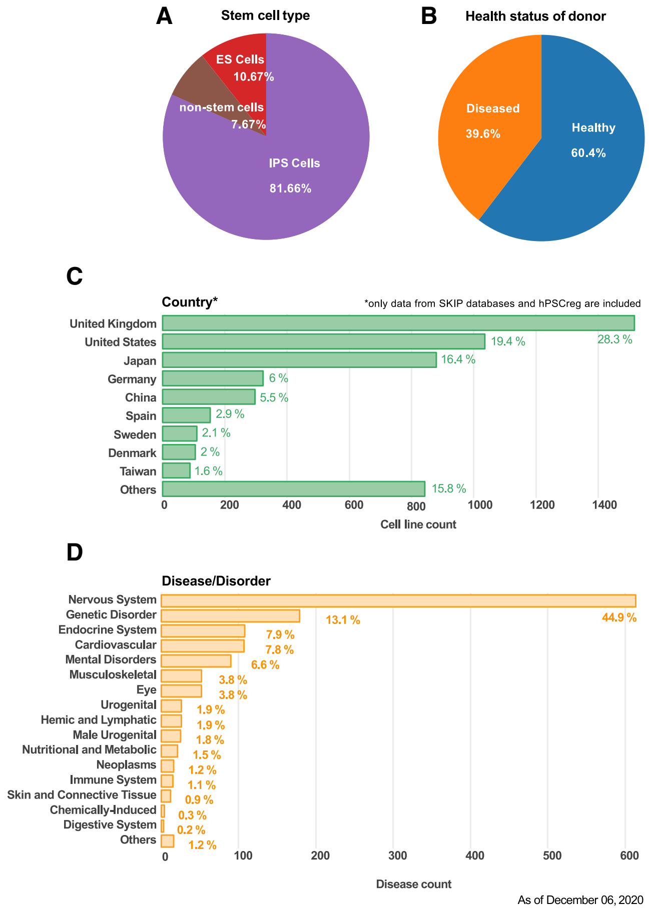

Figure 2. Details of Cell Lines Collected by ICSCB

Cell line information is categorized as (A) stem cell type, (B) health/disease status of donor, (C) country that established the cell lines, and (D) disease category. ES cells, embryonic stem cells.

allows us to check the current state of stem cell research in the world (Figure 2). Although we recognize redundancies in the data, according to our statistics, iPSC lines constitute more than 80% of all cell lines, and the ratio of healthy to diseased donors is approximately 3 to 2 (Figures 2A and 2B). The total number of countries from which cell lines can be retrieved is 39 (as of December 6, 2020), of which the top 9 countries identified in SKIP and hPSCreg are (in descending order) the United Kingdom, the United States, Japan, Germany, China, Spain, Sweden, Denmark, and Taiwan (Figure 2C). In addition, as the number of iPSC lines generated from patient donors has been growing recently, ICSCB supports disease-oriented searches to help users find all disease-related stem cell lines by using disease names. The distribution of disease and disorder types is shown in Figure 2D.

Easy Search Interface on SHOGoiN Homepage

ICSCB also has a quick and easy search module on the SHOGoiN homepage (https://stemcellinformatics.org/). SHOGoiN is a repository for accumulating and integrating diverse human cell information to support a wide range of research using cell-related data. The database consists of several modules that store cell lineage maps, transcriptomes, methylomes, cell conversions, cell type markers, and cell images with morphology data curated from public as well as contracted resources based on sophisticated cell taxonomy. Collaboration between ICSCB and SHOGoiN makes it possible for users to directly use free text searches for stem cell line data on the SHOGoiN homepage. The ICSCB easy search module in SHOGoiN supports a simplified ICSCB search with keywords, and the advanced search is designed to redirect users to the ICSCB homepage with full functions. Results from the SHOGoiN homepage have the same structure as in the ICSCB homepage.

DISCUSSION

Concluding Remarks and Future Plan

So far, the registration and submission of newly established cell lines have been complicated by the lack of standardized data formats. Most data registries are currently limited by respective domestic policies and have adopted their information structures and validation processes independently (Andrews et al., 2015; Zarzeczny et al., 2009). The lack of standardized data formats has caused problems for researchers, who must usually search several websites to find the stem cell lines they are looking for (Wells et al., 2013). In the present work, we developed ICSCB, an integrated data distribution system that provides stem cell line information from major stem cell banks and registries all over the world. ICSCB adopts a standardized information format based on the “Source Cell” module of MIACARM to integrate different data resources while keeping important information.

ICSCB has several limitations; for instance, there exist cell lines having limited donor information and/or incomplete information, as well as replicates of the same line with different names. In addition, ICSCB contains only a few clinical-grade cells due to strict requirements for exhaustive as well as expensive quality checks and haplotype compatibility for clinical-grade lines. Currently, when searching for stem cell lines for a specific disease, users have limited access to a distinct aspect of research for the diseases registered in individual repositories. Indeed, our next step would be to establish an integrated and refined collection of research on stem cell lines in order to understand the possible causes and mechanisms of complex diseases on the basis of genetic background and environmental effects in terms of molecular pathways during the developmental process. We may need a new project based on new funding to establish such a global collaboration.

ICSCB has several issues that merit improvement. First, we plan to assign unique accession codes to all cell line entries by

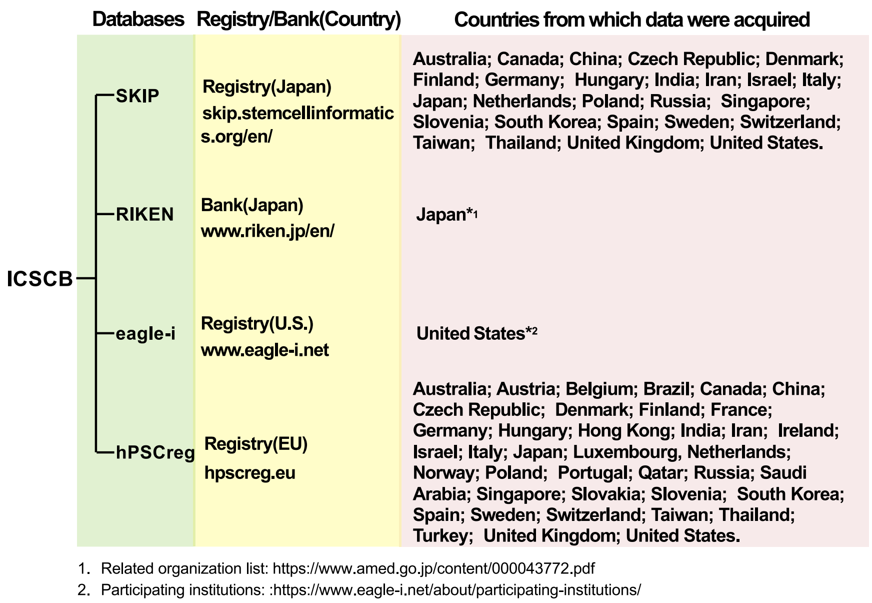

Figure 3. Overview of ICSCB
ICSCB includes data from three stem cell registries and one cell bank in order to maximize data coverage worldwide.

utilizing standardized nomenclature for pluripotent stem cells (Kurtz et al., 2018) in order to remove redundant cell line data. Second, as the cost of experimental technologies, such as genome/RNA sequencing or teratoma assay, to characterize stem cell lines decreases and it becomes easy to obtain various types of profiles on them, we may be able to

Table 2. Cross-Reference Table for Integration of Four Databases according to MIACARM Module (as of December 6, 2020)

MIACARM Module	ICSCB Term	hPSCreg	SKIP	RIKEN BRC	eagle-i
Stem cell general identification	*Stem cell ID*	stem cell id	stem cell id	CellID	cell line label
	Stem cell name	stem cell name	cell line name	CellName	cell line label
	Stem cell type	NA	cell type	cell grouping	cell line type
	Cell grade	NA	research grade	NA	NA
	Produced by	produced by	establisher name	originator	NA
	Provider/distributor	distributor	establisher organization	depositor	cell line provider
	Reference publication	publication	pubmed ID	reference	NA
Donor identification	*Gender of donor*	gender	donor sex	gender	sex
	Ethnicity of donor	race	donor race	race	ethnicity
	Health status	health status	disease name	disease	diagnosed disease
Source cell identification	*Source cell type*	source cell type	NA	NA	NA
	Organ/tissue of origin of source cell	origin of source cell	organ/tissue of origin of source cell	NA	NA
Ethical operation	*Informed consent from donor*	NA	NA	description	NA
	MTA	NA	NA	description	NA
Data	*Data ID*	NA	NA	NA	NA
	Data type	NA	NA	NA	NA

MTA, material transfer agreement; NA, not available.

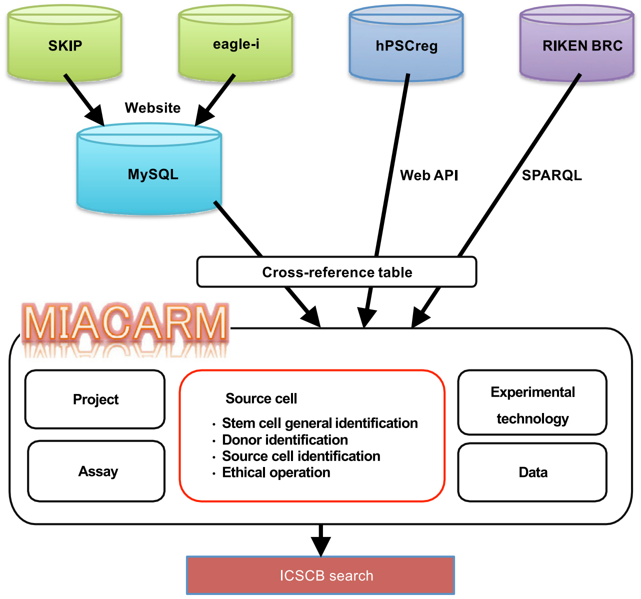

Figure 4. Workflow of ICSCB Data Integration
The SKIP and eagle-i databases were fully replicated from websites and imported to MySQL, whereas hPSCreg and RIKEN BRC used a web API and SPARQL for data collection, respectively. A cross-reference table (Table 2) was used when ICSCB integrated and standardized cell line data.

define a standard profile set for a complete data format to render comparisons of cell lines more efficient. Third, once the RNA-sequencing or genome mutation data are collected, it will be possible to perform statistical analysis such as principal component analysis or other refined bioinformatics methods to mathematically map individual cell lines to a global stem cell feature space, such as differentiation propensity, carcinogenic potential, immune response, and so on. We expect ICSCB to further evolve, thereby providing users better accessibility to relevant stem cell lines.

In the future, to respond to the rapid growth in the number of stem cell lines, we will include more data resources in ICSCB, including the Taiwan Human Disease iPSC Service Consortium and other recently developed stem cell banks, to make ICSCB more resource abundant and usable. We also plan to add a detailed quality check to help users find stem cell lines of high quality. As the largest stem cell line information resource, we will support stem cell communities by improving the quality and increasing the scale of our database.

EXPERIMENTAL PROCEDURES

Data Resources

ICSCB resources were selected from existing major stem cell registries that collect cell line information in Europe, Japan, and the United States and stem cell banks that provide cell lines with information on the attributes. We checked the number of registered cell lines and the criteria for registration in these registries and banks to decide to what extent their cell line data can fulfill MIACARM guidelines for inclusion in ICSCB. Considering the size, accessibility, and diversity of the different databases, three stem cell registries and one stem cell bank were included: (1) SKIP (5,615 cell lines), (2) hPSCreg (3,360 cell lines), (3) RIKEN BRC (3,548 cell lines), and (4) eagle-i (3,548 cell lines) (as of December 6, 2020). These data resources were selected because they had the highest number of registered cell lines and a large diversity, which would provide a good regional balance of cell sources to reduce redundancies in cell line entries. RIKEN BRC basically collected cell lines from Japanese institutions, SKIP contained data mostly from other Japanese and Asian institutions, hPSCreg collected data mainly from European institutions, and eagle-i collected data mostly from the United States. Details of the data sources are listed in Figure 3.

Data Integration

Since our previous research on the listed stem cell banks 4 years ago (Sakurai et al., 2016), the number of registered cell lines has skyrocketed, from 1,483 to approximately 8,000. As a result, stem cell registries are tasked with collecting information on the rapidly increasing number of new cell lines and registering the cell lines into their databases as quickly as possible. However, because the stem cell banks and registries are using their own formats for data entry, the integration of the data into a centralized collection system is an extraordinary challenge. To solve this problem, we used a decentralized or distributed database system (Fujibuchi et al., 1998) by adopting items of different database formats into 16 attributes, or terms, from three MIACARM modules: stem cell general identification, donor identification, and source cell identification (Table 2). To practically integrate the data from the four data resources (SKIP, hPSCreg, RIKEN BRC, and eagle-i), we adopted a mechanism of cross-reference tables that allow users to conduct a search using MIACARM terms that are translated into the corresponding terms in the individual data resources to implement the search. For example, the term “Stem cell ID” in MIACARM was translated into the terms “stem cell id” (hPSCreg), “stem cell id” (SKIP), “CellID” (RIKEN BRC), and “cell line label” (eagle-i) for the search implementation. Thus, ICSCB submits search requests to each data resource with its own (translated) terms and integrates all retrieved results by common MIACARM terms, thereby achieving a standardized data format at the level of display (Figure 4).

ICSCB workflow and search engine updating

To provide fast and easy access to the latest and accurate cell line information, we built an automatic updating system that adds newly released cell lines to ICSCB as soon as they become available in any of the four data resources. Data from eagle-i and SKIP are directly collected and stored in the MySQL database with the terms required for the MIACARM modules. Data from hPSCreg and RIKEN BRC are collected on the fly per request using a web application programming interface (API) provided by the respective

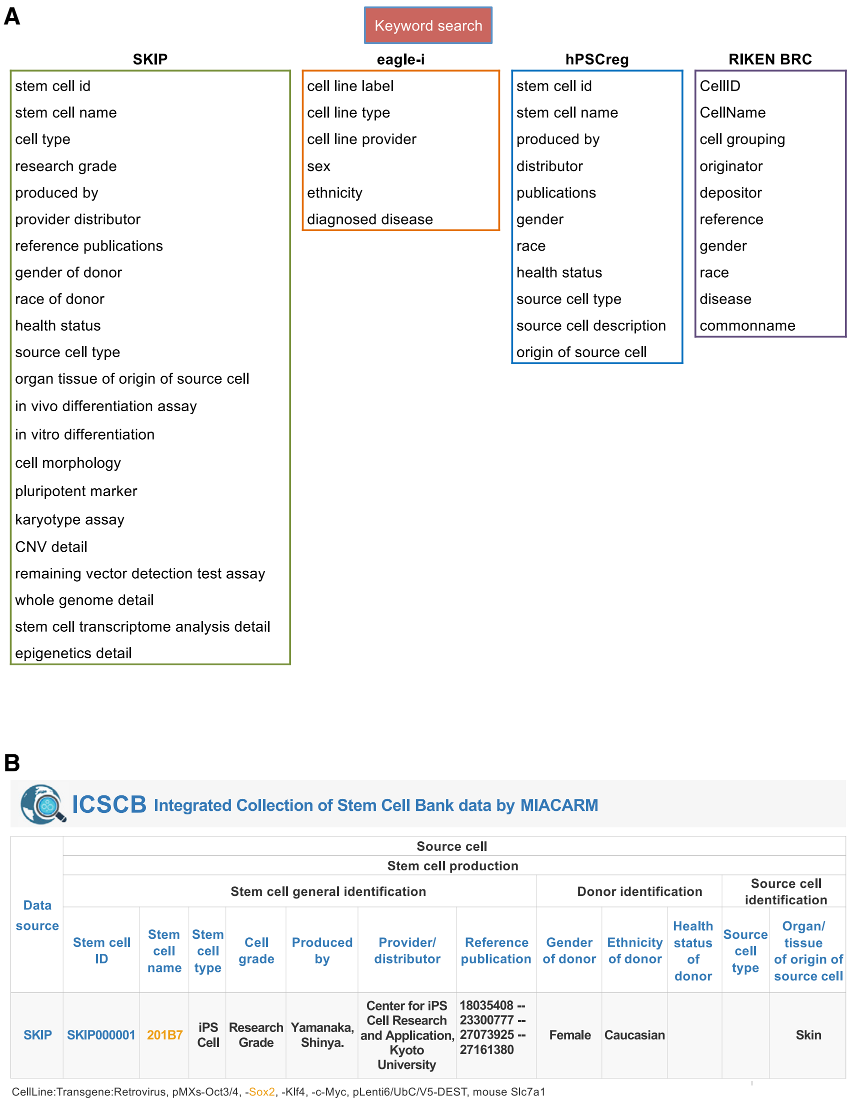

Figure 5. Keyword Search Is Automatically Extended to All Terms Provided by the Four Data Resources, Even if a Keyword Is Not Included in Standardized MIACARM Terms

(A) Terms specific to each of the four data resources.

(B) Even if the standardized MIACARM terms do not contain, for example, “transgene,” it is still possible to enter a gene name into the keyword field (e.g., *SOX2*), which will lead users to results from the four data resources with relevant information. The results of the match will be shown in another row below the standardized fields.

sites. RIKEN BRC also uses SPARQL language for data retrieval requests (Kim et al., 2017; Kobayashi et al., 2016).

To simplify the search process, ICSCB provides an easy-to-use and mobile-friendly web application. The goal of the application is to help users find the desired stem cell lines as quickly as possible. The interface of the search engine is designed with the 16 MIACARM terms (Table 2), except the term “Stem cell ID.” Users receive result pages containing all the matching results listed in a table that includes all the basic attributes under the structure of MIACARM. To ensure a more specific search with a wide variety of attributes, ICSCB is designed to accommodate searches not only by standardized terms from MIACARM but also by terms specific to each of the four data resources, such as “age” or “country” (Figure 5A). When user queries are submitted, ICSCB simultaneously retrieves MIACARM standardized data and resource-specific data so as not to miss any relevant entries. If a keyword entered by a user in a general keyword search does not exist in MIACARM terms, but is included in data specific to any of the four data resources, the user will get detailed descriptions of the matching data in the results page. For example, even if the standardized MIACARM terms do not contain “transgene,” it is still possible to enter a gene name into the keyword field (e.g., *SOX2*) such that the results page will display relevant entries by showing the indicated keyword in the extra field below (Figure 5B). Furthermore, the user can filter the results by data resource and detailed keywords from the “Searching options” box on the results page to narrow down the results list. In addition, all results can be easily downloaded as a table directly from the results page.

ICSCB also provides a quality control panel based on MIACARM, thereby supporting customized searches according to quality control results. At present, assays for teratoma formation,

differentiation ability *in vitro*, morphology data, marker gene expression/surface antigen expression data, karyotyping assay results, copy number variation, residual exogene detection results, genome profiling, transcriptome profiling, and epigenome profiling data are accessible from ICSCB.

Data and Code Availability

The original data and R source code for creating figures and supplementary figures and tables are available at https://github.com/YingChen-bio/ICSCB.

SUPPLEMENTAL INFORMATION

Supplemental information can be found online at https://doi.org/10.1016/j.stemcr.2021.02.014.

AUTHOR CONTRIBUTIONS

Y.C. and Y.P. drafted the manuscript. S.M., T.M., H.O., J.D., S.S., A.K., H.M., Y.N., M.S., J.S., and W.F. provided and facilitated the stem cell data. W.F. and K.S. conceptualized the research. A.K., G.S., and W.F. led the project.

CONFLICTS OF INTEREST

H.O. is a founding scientist of SanBio Co., Ltd., and K Pharma, Inc.

ACKNOWLEDGMENTS

The authors deeply appreciate Dr. Peter Karagiannis for kindly reviewing the manuscript. This work was partially supported by the Core Center for iPS Cell Research, Research Center Network for Realization of Regenerative Medicine (16bm0104001h0004) and the Formulation of Regenerative Medicine National Consortium, Which Renders Nation-wide Assistance to Clinical Researches, Project to Build Foundation for Promoting Clinical Research of Regenerative Medicine (19bk0204001h0004, 19bk0204001s0104), the Japan Agency for Medical Research and Development (AMED); and a German Academic Exchange Service (DAAD) PPP grant.

Received: April 8, 2020
Revised: February 16, 2021
Accepted: February 18, 2021
Published: March 18, 2021

REFERENCES

Andrews, P.W., Baker, D., Benvinisty, N., Miranda, B., Bruce, K., Brüstle, O., Choi, M., Choi, Y.-M., Crook, J.M., and Dvorak, P. (2015). Points to consider in the development of seed stocks of pluripotent stem cells for clinical applications: international Stem Cell Banking Initiative (ISCBI). Regen. Med. *10*, 1–44.

Fujibuchi, W., Goto, S., Migimatsu, H., Uchiyama, I., Ogiwara, A., Akiyama, Y., and Kanehisa, M. (1998). DBGET/LinkDB: an integrated database retrieval system. Pac. Symp. Biocomput. *98*, 683–694.

Godard, B., Schmidtke, J., Cassiman, J.J., and Aymé, S. (2003). Data storage and DNA banking for biomedical research: informed consent, confidentiality, quality issues, ownership, return of benefits. A professional perspective. Eur. J. Hum. Genet. *11*, S88–S122.

Hug, K. (2009). Banks, repositories and registries of stem cell lines in Europe: regulatory and ethical aspects. Stem Cell Rev. Rep. *5*, 18–35.

Isasi, R., and Knoppers, B.M. (2011). From banking to international governance: fostering innovation in stem cell research. Stem Cells Int. *2011*, 498132.

Kim, J.H., Kurtz, A., Yuan, B.Z., Zeng, F., Lomax, G., Loring, J.F., Crook, J., Ju, J.H., Clarke, L., Inamdar, M.S., et al. (2017). Report of the International Stem Cell Banking Initiative workshop activity: current hurdles and progress in seed-stock banking of human pluripotent stem cells. Stem Cells Transl. Med. *6*, 1956–1962.

Knoppers, B.M., and Isasi, R. (2010). Stem cell banking: between traceability and identifiability. Genome Med. *2*, 73.

Kobayashi, N., Lenz, K., and Masuya, H. (2016). RIKEN MetaDatabase: a database platform as a microcosm of linked open data cloud in the life sciences. In Joint International Semantic Technology Conference (Springer), pp. 99–115.

Kurtz, A., Seltmann, S., Bairoch, A., Bittner, M.S., Bruce, K., Capes-Davis, A., Laurence, D., Johannes, D., Clarke, L., Crook, J.M., et al. (2018). A standard nomenclature for referencing and authentication of pluripotent stem cells. Stem Cell Reports *10*, 1–6.

Nishizawa, M., Chonabayashi, K., Nomura, M., Tanaka, A., Nakamura, M., Inagaki, A., Nishikawa, M., Takei, I., Oishi, A., Tanabe, K., et al. (2016). Epigenetic variation between human induced pluripotent stem cell lines is an indicator of differentiation capacity. Cell Stem Cell *19*, 341–354.

Sakurai, K., Kurtz, A., Stacey, G., Sheldon, M., and Fujibuchi, W. (2016). First proposal of minimum information about a cellular assay for regenerative medicine. Stem Cells Transl. Med. *5*, 1345–1361.

Sasaki, K., Makiyama, T., Yoshida, Y., Wuriyanghai, Y., Kamakura, T., Nishiuchi, S., Hayano, M., Harita, T., Yamamoto, Y., Hirose, S., et al. (2016). Patient-specific human induced pluripotent stem cell model assessed with electrical pacing validates S107 as a potential therapeutic agent for catecholaminergic polymorphic ventricular tachycardia. PLoS One *11*, e0164795.

Seltmann, S., Lekschas, F., Müller, R., Stachelscheid, H., Bittner, M.S., Zhang, W., Luam, K., Anna, S., Anna, V., Stacey, G.N., et al. (2015). hPSCreg—the human pluripotent stem cell registry. Nucleic Acids Res. *44*, D757–D763.

Takahashi, K., Tanabe, K., Ohnuki, M., Narita, M., Ichisaka, T., Tomoda, K., and Yamanaka, S. (2007). Induction of pluripotent stem cells from adult human fibroblasts by defined factors. Cell *31*, 861–872.

Thirumala, S., Goebel, W.S., and Woods, E.J. (2009). Clinical grade adult stem cell banking. Organogenesis *5*, 143–154.

Vasilevsky, N., Johnson, T., Corday, K., Torniai, C., Brush, M., Segerdell, E., Wilson, M., Shaffer, C., Robinson, D., and Haendel,

M. (2012). Research resources: curating the new eagle-i discovery system. Database *2012*, bar067.

Wells, C.A., Mosbergen, R., Korn, O., Choi, J., Seidenman, N., Matigian, N.A., Vitale, A.M., and Shepherd, J. (2013). Stemformatics: visualisation and sharing of stem cell gene expression. Stem Cell Res. *10*, 387–395.

Winickoff, D.E., Saha, K., and Graff, G.D. (2009). Opening stem cell research and development: a policy proposal for the management of data, intellectual property, and ethics. Yale J. Health Policy Law Ethics *9*, 52–127.

Yaffe, M.P., Noggle, S.A., and Solomon, S.L. (2016). Raising the standards of stem cell line quality. Nat. Cell Biol. *18*, 236.

Zarzeczny, A., Scott, C., Hyun, I., Bennett, J., Chandler, J., Chargé, S., Heine, H., Isasi, R., Kato, K., Lovell-Badge, R., et al. (2009). iPS cells: mapping the policy issues. Cell *139*, 1032–1037.

Stem Cell Reports, Volume *16*

Supplemental Information

Integrated Collection of Stem Cell Bank Data, a Data Portal for Standardized Stem Cell Information

Ying Chen, Kunie Sakurai, Sumihiro Maeda, Tohru Masui, Hideyuki Okano, Johannes Dewender, Stefanie Seltmann, Andreas Kurtz, Hiroshi Masuya, Yukio Nakamura, Michael Sheldon, Juliane Schneider, Glyn N. Stacey, Yulia Panina, and Wataru Fujibuchi

SUPPLEMENTAL FIGURES

Fig S1. Related to Figure 2.

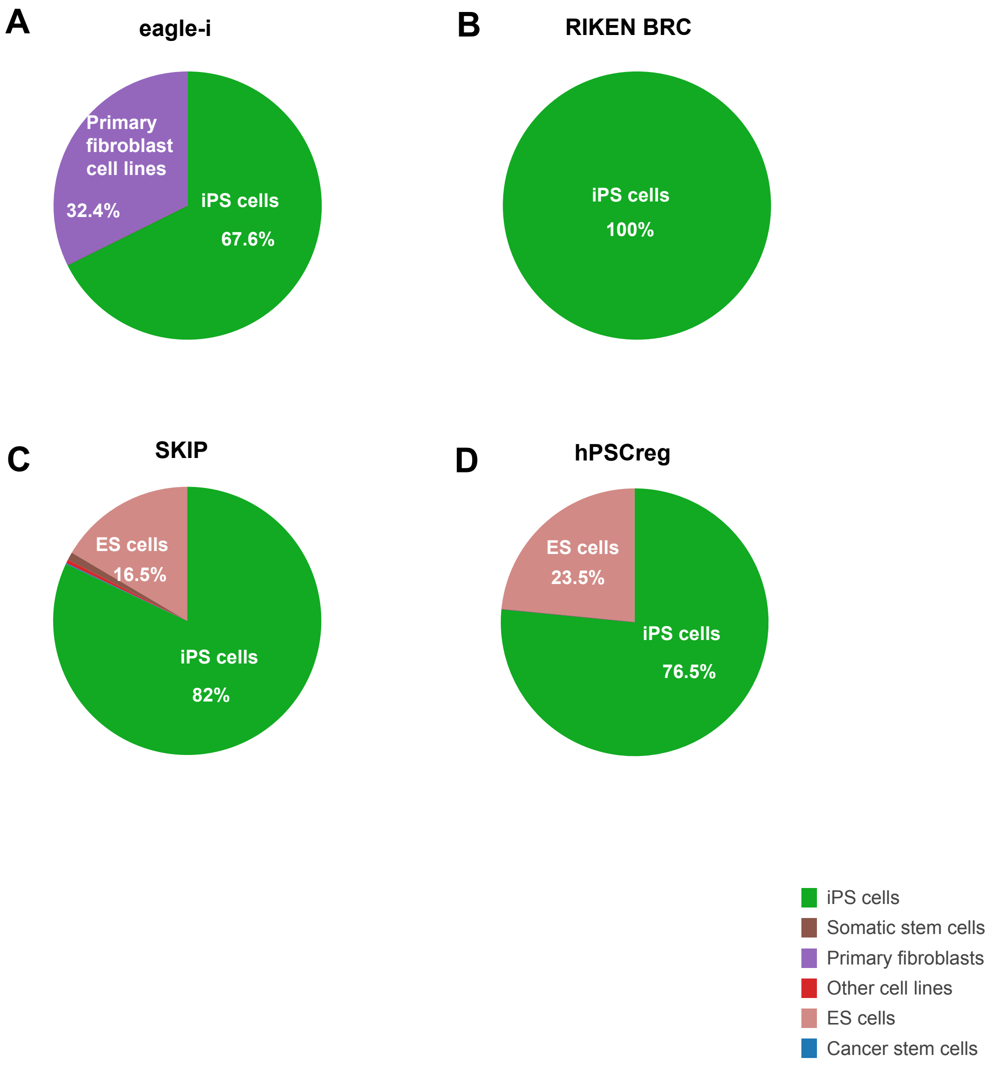

Fig S1. Details of cell line types collected by eagle-i, RIKEN BRC, SKIP, and hPSCreg (as of December 6, 2020)**.** (A) eagle-i, (B) RIKEN BRC, (C) SKIP, and (D) hPSCreg.

Fig S2. Related to Figure 2.

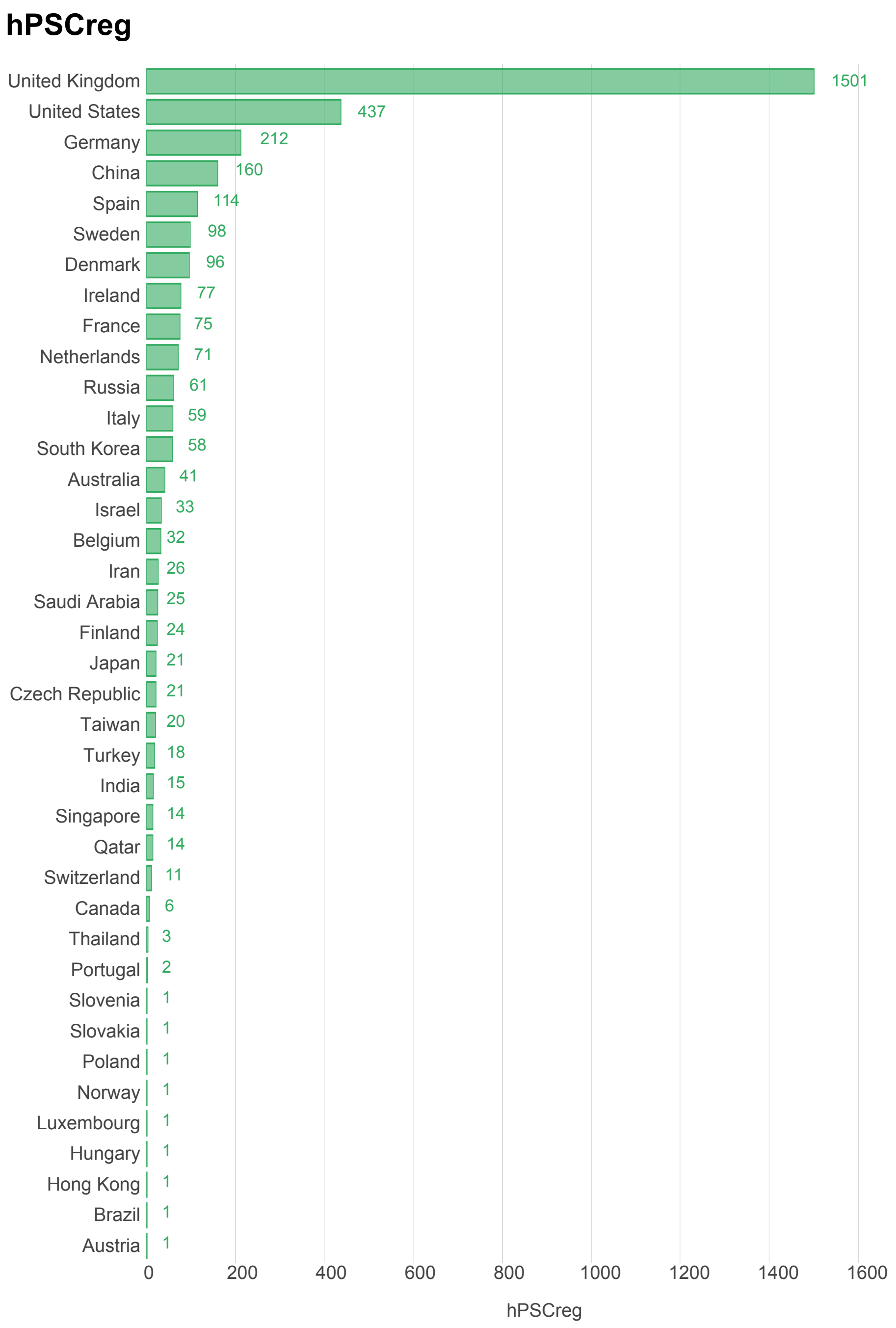

Fig S2. **Details of countries that have established cell lines in hPSCreg** (as of December 6, 2020).

Fig S3. Related to Figure 2.

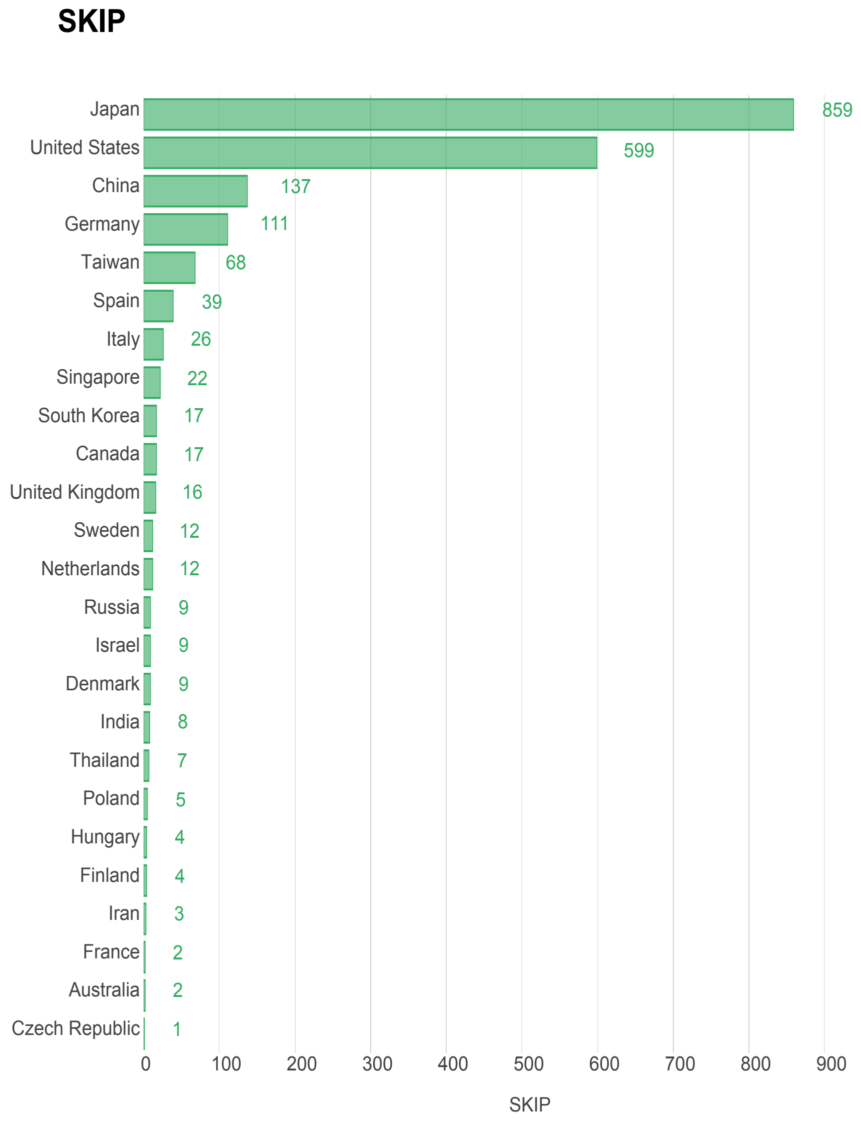

Fig S3. Details of countries that have established cell lines in SKIP (as of December 6, 2020)**.**

SUPPLEMENTAL TABLES

Table S1. Related to Figure 5. (submitted as separate Excel file)

Table S1. List of full information of ICSCB (as of December 6, 2020)**.**

Table S2. Related to Figure 2A and Figure S1.

Stem_cell_type	hPSCreg	SKIP	RIKEN BRC
**ES cells**	788	927	0
**iPS cells**	2572	4603	3548
**Somatic stem cells**	0	54	0
**Cancer stem cells**	0	7	0
**Primary fibroblast cell lines**	0	0	0
**Others**	0	24	0
**Total**	**3360**	**5615**	**3548**

Table S2. List of cell line types across all four databases (as of December 6, 2020)**.**

Table S3. Related to Figure 2B.

Health_status	Count
Healthy	9708
Diseased	6363
**Total**	**16071**

Table S3. Statistics of healthy/diseased cell lines in ICSCB (as of December 6, 2020)**.**

Table S4. Related to Figure 2C.

Country	SKIP	hPSCreg	Total
**United Kingdom**	16	1501	1517
**United States**	599	437	1036
**Japan**	859	21	880
**Germany**	111	212	323
**China**	137	160	297
**Spain**	39	114	153
**Sweden**	12	98	110
**Denmark**	9	96	105
**Taiwan**	68	20	88
**Italy**	26	59	85
**Ireland**	0	77	77
**South Korea**	17	58	75
**Netherlands**	12	71	83
**Russia**	9	61	70
**France**	2	75	77
**Australia**	2	41	43
**Israel**	9	33	42
**Singapore**	22	14	36
**Belgium**	0	32	32
**Iran**	3	26	29
**Canada**	17	6	23
**Finland**	4	24	28
**Czech Republic**	1	21	22
**India**	8	15	23
**Turkey**	0	18	18
**Qatar**	0	14	14
**Switzerland**	0	11	11
**Thailand**	7	3	10
**Hungary**	4	1	5
**Poland**	5	1	6
**Portugal**	0	2	2
**Saudi Arabia**	0	25	25
**Austria**	0	1	1
**Brazil**	0	1	1
**Luxembourg**	0	1	1
**Norway**	0	1	1
**Slovakia**	0	1	1
**Slovenia**	0	1	1
**Hong Kong**	0	1	1
**Total**	**1998***	**3354****	**5352**

*The number was calculated by 5615 (total) - 3647 (hPSCreg or eagle-i or unknown country) + 30 (dual country) = 1998.

**The number was calculated by 3360 (total) - 6 (synonyms) = 3354

Table S4. Statistics of cell line types based on country (as of December 6, 2020)**.**

Table S5. Related to Figure 2D.

Disease Category in MeSH	Count of diseases
Urogenital Diseases	26
Skin and Connective Tissue Diseases	12
Others	16
Nutritional and Metabolic Diseases	21
Nervous System Diseases	614
Neoplasms	16
Musculoskeletal Diseases	52
Mental Disorders	90
Male Urogenital Diseases	25
Immune System Diseases	15
Hemic and Lymphatic Diseases	26
Genetic Disorders	179
Eye Diseases	52
Endocrine System Diseases	108
Digestive System Diseases	3
Chemically Induced Disorders	4
Cardiovascular Diseases	107
**Total**	**1366**

Table S5. Statistics of diseased cell lines based on disease category (as of December 6, 2020)**.**

EXPERIMENTAL PROCEDURES

Generation of Fig. 1

Among all the databases, SKIP and hPSCreg provided details of countries from which data were acquired. For SKIP, this information was provided on its homepage (skip.stemcellinformatics.org/en/). For hPSCreg, country information for every cell line could be accessed from its homepage (https://hpscreg.eu/) by clicking “find by location”.

Generation of Fig. 2

Full information data were directly downloaded from ICSCB results page (Table S1) and filtered according to the following criteria: (A) stem cell type (Table S2); (B) health/disease status (Table S3); (C) country (Table S4); and (D) disease (Table S5). Disease categories were determined by search results with keywords under the “Disease Category” in NCBI MeSH page (https://www.ncbi.nlm.nih.gov/mesh). For example, searching with keywords of “Parkinson disease” will lead to the MeSH term “Nervous System Diseases” under the “Disease Category”. Pie charts and bar graphs were produced by R (graph.r) using the package “plotly”.
